# Anlotinib in the treatment of advanced hepatocellular carcinoma: an open-label phase II study (ALTER-0802 study)

**DOI:** 10.1007/s12072-021-10171-0

**Published:** 2021-04-07

**Authors:** Yongkun Sun, Aiping Zhou, Wen Zhang, Zhichao Jiang, Bo Chen, Jianjun Zhao, Zhiyu Li, Liming Wang, Xinyu Bi, Hong Zhao, Kan Liu

**Affiliations:** 1 Department of Medical Oncology, National Cancer Center/National Clinical Research Center for Cancer/Cancer Hospital, Chinese Academy of Medical Sciences and Peking Union Medical College, Beijing, 100021 People’s Republic of China; 2 Department of Radiotherapy, National Cancer Center/National Clinical Research Center for Cancer/Cancer Hospital, Chinese Academy of Medical Sciences and Peking Union Medical College, Beijing, 100021 People’s Republic of China; 3 Department of Hepatobiliary Surgery, National Cancer Center/National Clinical Research Center for Cancer/Cancer Hospital, Chinese Academy of Medical Sciences and Peking Union Medical College, Beijing, 100021 People’s Republic of China; 4 Department of Medical Imaging, National Cancer Center/National Clinical Research Center for Cancer/Cancer Hospital, Chinese Academy of Medical Sciences and Peking Union Medical College, Beijing, 100021 People’s Republic of China

**Keywords:** Hepatocellular carcinoma, Anlotinib, Anti-tumor efficacy, Adverse events, Biomarker, Cytokine, Tyrosine kinase inhibitors, Progression-free survival, Time to progression, Safety

## Abstract

**Purpose:**

This study aimed to assess efficacy and safety of anlotinib as a first- or second-line treatment for advanced or metastatic hepatocellular carcinoma (aHCC) and to identify the predictive plasma cytokines on efficacy of anlotinib.

**Methods:**

It was a phase II clinical study. Patients with aHCC were recruited from October 2016 to April 2019 and divided into two cohorts according to previous tyrosine kinase inhibitors (TKIs) therapy. Those without or with prior TKIs were in Cohort 1 or 2, respectively. All patients took anlotinib (12 mg/day, Day1–14, 3 weeks per cycle). The primary endpoint was 12-week progression-free survival (PFS) rate. Relationship between the series plasma cytokine level and the efficacy of anlotinib was analyzed.

**Results:**

Enrolled 26 patients in Cohort 1 and 24 in Cohort 2. In Cohort 1, the 12-week PFS rate was 80.8% [95% confidence interval (CI); 59.8%–91.5%] and median time to progression (TTP) was 5.9 months (95% CI 4.8–6.9). In Cohort 2, the 12-week PFS rate and median TTP was 72.5% (95% CI 48.7%–86.6%) and 4.6 months (95% CI 2.7–10.0), respectively. The median TTP on patients with a baseline plasma level of CXCL1 (C-X-C motif chemokine ligand 1) less than 7.6 ng/μl was significantly longer in both cohorts. The most common grade 3–5 adverse events were hypertension (8%), diarrhea (8%) and hand-foot syndrome (6%).

**Conclusion:**

Anlotinib showed promising efficacy and safety as a first- or second-line treatment with a continuous TKIs treatment strategy in aHCC. The plasma CXCL1 might be a predictor for the efficacy of anlotinib.

**Supplementary Information:**

The online version contains supplementary material available at 10.1007/s12072-021-10171-0.

## Introduction

Hepatocellular carcinoma (HCC) ranks the seventh most common and second most fatal malignant tumor worldwide [1]. Half of the prevalence and mortality occurred in China due to the high incidence of Hepatitis B Virus infection [2]. Angiogenesis-based targeted therapy has been standard management for advanced HCC since the approval of sorafenib in 2007. Though there was a limited survival benefit, especially in Chinese patients, with a median time to progression (TTP) of 2.8 months and overall survival (OS) of 6.5 months in a pivotal Oriental trials, sorafenib remained the unique targeted agent in first-line treatment until the approval of lenvatinib in 2019 [3]. Three other agents, regorafenib, carbozanitinib and ramucirumab, were approved for second-line treatment, and progression-free survival (PFS) varied from 2.8 to 5.2 months and OS from 8.5 to 10.6 months [4–6]. It suggested the importance of continuous anti-angiogenic treatment for HCC.

Several issues on angiogenesis targeted therapy remain clinically relevant for HCC. First of all, resistance occurs inevitably. Though the exact mechanism of resistance remains unclear, it is believed the activation of the Fibroblast Growth Factor Receptor (FGFR) pathway, another angiogenic pathway, plays an important role [7]. Secondly, no predictive biomarkers have been clearly defined for angiogenesis-based therapy. A Japanese study found a relationship between the better efficacy of lenvatinib and a higher level of serum Fibroblast growth factor 19 (FGF19) or a lower level of Angiopoietin 2 (Ang-2) [8]. However, the markers have not been tested in large-scale studies.

The approval of Nivolumab in September 2017 unveiled a new era of immunotherapy for HCC. But it failed to show superiority to sorafenib as a first-line treatment. The embarrassment was terminated by IMbrave 150 study. The combination of Atezolizumab (Programmed Death Ligand-1 [PD-L1] inhibitor) and bevacizumab achieved significant improvements over sorafenib in median OS (19.2 vs. 13.4 months, *p* = 0.0009), PFS (6.9 vs. 4.3 months, *p* = 0.0001) and ORR (30% vs. 11%) [9]. While consistent activities are exhibited with other combinations of immune checkpoint inhibitors (ICIs) and different anti-angiogenic Tyrosine Kinase Inhibitors (TKIs), the safety profile has become more clinically concerning. Better tolerated TKIs are sought to be used in combination with PD-1/PD-L1 inhibitors. Overall, it is still vital to develop novel potent targeted agents with favorable safety profiles and abilities to overcome anti-angiogenesis resistance.

Anlotinib is a novel multi-targeting TKI, with substantial inhibitory activity against Vascular Endothelial Growth Factor Receptor (VEGFR) 1–3, Platelet-derived Growth Factor Receptor (PDGFR) α/β, FGF Receptor 1–4, and c-kit [10]. In vitro studies, anlotinib blocked VEGFR2 with an extremely low half-maximal inhibitory concentration (IC50) value of 0.2 nM [11]. Anlotinib downregulated phosphorylation of FGFR1 at an inhibition rate of 45.0% (p-FGFR1/FGFR1) at 1 μM, and showed an IC50 value of 25 nM in AN3Ca cells overexpressing mutant FGFR2 [11, 12]. Anlotinib has been approved for the treatment of non-small cell lung cancer, small cell lung cancer and soft tissue sarcoma in China. Due to its potent inhibitory activity and additional activity against FGFR2, anlotinib was believed to have favorable antitumor activity as first- or second-line treatment in HCC. In vitro studies, anlotinib significantly inhibited the proliferation of HCC cells and prompted apoptosis. Subsequent animal experiments also illustrated that anlotinib reduced HCC progression [13]. To clarify the clinical efficacy and safety of anlotinib in first- and second-line treatment for advanced HCC, we conducted this pilot phase II study (NCT02809534) since October 2016, when sorafenib stood as the unique available targeted agent worldwide. We also analyzed the plasma level of some concerned cytokines with an attempt to identify the predictive markers.

## Patients and methods

### Study design

This was an open-label phase II clinical trial conducted at the Cancer Hospital of the Chinese Academy of Medical Sciences. It was carried out in accordance with the Declaration of Helsinki and Good Clinical Practice Guidelines and was approved by the independent institutional ethics committee. All enrolled patients signed written informed consent forms.

### Patients

Patients enrolled were 18–75 years old and had locally advanced or metastatic HCC with a histologically or cytologically confirmed diagnosis. Eligible patients were ineligible for or refractory to locoregional therapies, including arterially directed therapies, ablation, radiotherapy and surgery; those were with Child–Pugh score < 8, Eastern Cooperative Oncology Group Performance Status (ECOG PS) 0 or 1; at least one measurable lesion according to Response Evaluation Criteria in Solid Tumors (RECIST) version 1.1; adequate vital organ function. The main exclusion criteria were a diagnosis of cholangiocarcinoma, mixed cell carcinoma or fibrolamellar cell carcinoma; HBV DNA > 2000 IU/ml; previous or preparation for liver transplantation; bleeding tendency; and brain metastasis.

Patients without previous TKIs treatment were in Cohort 1, while those had been treated by prior first-line TKIs were in Cohort 2.

### Procedures

Anlotinib were given orally once daily at 12 mg on Day 1–14, every three weeks per cycle. The treatment continued until progressive disease (PD) or intolerable toxicity. Dosage reduction to 10 mg/day or even 8 mg/day of anlotinib was allowed due to grade 3 non-hematologic or grade 4 hematologic toxicities.

Scheduled visits and computed tomography (CT) or magnetic resonance imaging (MRI) examinations were performed every two cycles. Tumor response was evaluated by investigators according to RECIST version 1.1. The Common Terminology Criteria Adverse Events version 4.0 (CTCAE 4.0) was used for grading adverse events (AEs).

### Clinical outcomes

The primary endpoint was 12-week PFS rate. The secondary endpoints included TTP, ORR, OS, 24-week PFS rate and safety. The 12-week PFS rate and 24-week PFS rate was the percentage of patients who did not develop PD or died at week 12 or week 24, respectively. TTP referred to time from enrollment to PD. OS was defined as the time from enrollment to death from any cause. ORR was the percentage of patients with a confirmed complete or partial response.

### Biomarker detection

In this study, 8 ml of peripheral blood was scheduled to be collected during enrollment and at disease progression for each patient. The plasma concentrations of VEGF, PDGF, FGF-2, Programmed Death-1 (PD-1), C-X-C motif chemokine ligand 1 (CXCL1), Interleukin-2 (IL-2) and other cytokines were detected by Luminex^®^ MAGPIX system (cytokines list were shown in Supplementary Table 1). Cytokine concentrations changes between the time of enrollment and PD were calculated. The correlation between baseline level of cytokines and TTP or OS were analyzed, in which the cutoff value was selected according to the median concentration of each cytokine or regulatory receiver operating characteristic (ROC) curve.

### Statistical analysis

It was sought to demonstrate a 12-week PFS rate of 80% in Cohort 1 and 70% in Cohort 2. Compared with the historical 12-week PFS rate of 65% in first-line sorafenib and 12-week PFS rate of 50% in second-line regorafenib [4, 14, 15], 24 patients in Cohort 1 and 22 in Cohort 2 would be enrolled to achieve a 80% power with a two-sided 5% test. The accrual and follow-up period was estimated to be 12 months and 18 months, respectively. Considered a potential drop-out of approximately 10% of patients, a total of 27 patients in Cohort 1 and 24 in Cohort 2 will be recruited.

Efficacy and safety assessments were based on full analysis set (FAS) and safety analysis set (SS), respectively, which both included patients had received at least one dose of anlotinib. Kaplan–Meier (KM) analysis was used to estimate the median value and 95% CI of OS and TTP. For comparison between cytokine concentrations at baseline and those at disease progression, the Wilcoxon signed-rank test was used. The log-rank test was used to evaluate the relationship between cytokine concentrations at a different cutoff value and TTP or OS.

All the statistical tests were double-sided; *p* values ≤ 0.05 were considered statistically significant. SAS version 9.4 (SAS Institute Inc.) was used for all statistical analysis. NCSS_PASS version 15 was used for sample size calculating.

## Results

### Patients’ baseline characteristics and treatment

**Fig. 1:**
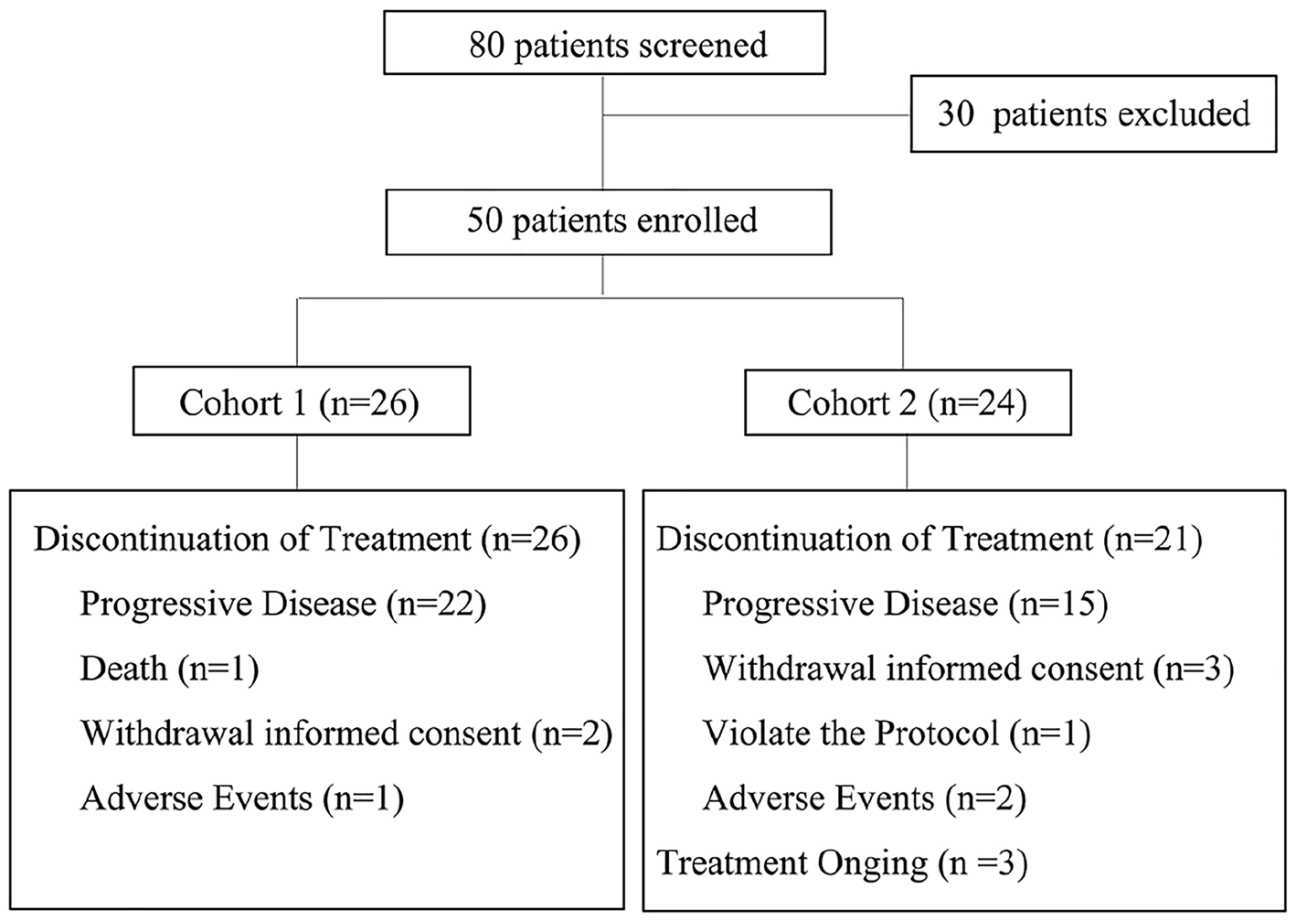
CONSORT diagram of two cohorts in this study. *RECIST* Response evaluation criteria in the solid tumor, *HBV* Hepatitis B virus, *DNA* Deoxyribonucleic acid, *AST* Aspartate aminotransferase, *ALT* Alanine aminotransferase, *TIBL* Total bilirubin, *PLT* Platelet count, *HGB* Hemoglobin

From October 2016 to April 2019, 80 patients were screened and 50 of them were enrolled. According to their previous treatment, 26 and 24 patients were in Cohort 1 and 2, respectively. By the cutoff date December 18, 2019, 1 patient in Cohort 1 and 3 in Cohort 2 were still on treatment (Fig. 1). All patients were included in the FAS and SS for analysis.

Baseline characteristics were shown in Table 1. The median duration of treatment was 5.0 months [95% CI confidence interval (CI); 4.4–5.6] and 4.1 months (95% CI 3.2–5.0) in two cohorts. Among all the patients, 14 accepted dose adjustment to 10 mg/day. There was no request to adjust to 8 mg/day.

**Table 1:** Baseline characteristics of the patients

	Total (*n* = 50)	Cohort 1 (*n* = 26)	Cohort 2 (*n* = 24)
Age, years, (Median, range)	53 (27–70)	53 (36–66)	54 (27–70)
Gender, *n* (%)			
Male	47 (94.0%)	25 (96.2%)	22 (91.7%)
Female	3 (6.0%)	1 (3.9%)	2 (8.3%)
ECOG PS, *n* (%)			
0	29 (58.0%)	14 (53.9%)	15 (62.5%)
1	21 (42.0%)	12 (46.2%)	9 (37.5%)
Child–Pugh score, *n* (%)			
5	34 (68.0%)	19 (73.1%)	15 (62.5%)
6	13 (26.0%)	7 (26.9%)	6 (25.0%)
7	3 (6.0%)	0	3 (12.5%)
BCLC stage, *n* (%)			
B	9 (18.0%)	5 (19.2%)	4 (16.7%)
C	41 (82.0%)	21 (80.8%)	20 (83.3%)
HBV status, *n* (%)			
Positive	40 (80.0%)	20 (76.9%)	20 (83.3%)
Negative	10 (20.0%)	6 (23.1%)	4 (16.7%)
HCV status, *n* (%)			
Positive	10 (20.0%)	5 (19.2%)	5 (20.8%)
Negative	40 (80.0%)	21 (80.8%)	19 (79.2%)
Previous Surgery, *n* (%)			
No	24 (48.0%)	11 (42.3%)	13 (54.2%)
Yes	26 (52.0%)	15 (57.7%)	11 (45.8%)
Macrovascular invasion, *n* (%)			
No	44 (88.0%)	23 (88.5%)	21 (87.5%)
Yes	6 (12.0%)	3 (11.5%)	3 (12.5%)
Extrahepatic metastasis, *n* (%)			
No	11 (22.0%)	7 (26.9%)	4 (16.7%)
Yes	39 (78.0%)	19 (73.1%)	20 (83.3%)
AFP, ng/ml, (Median, Range)	189.0 (1.8–441,231)	189.0 (1.8–82,630)	998.8 (2.4–441,231)
AFP ≥ 400 ng/ml	24 (48.0%)	12 (46.2%)	12 (50.0%)
AFP < 400 ng/ml	26 (52.0%)	14 (53.9%)	12 (50.0%)
Prior target therapy^a^, *n* (%)			
Yes		0 (0%)	24 (100%)

*ECOG PS* Eastern Cooperative Oncology Group Performance Status, *BCLC* Barcelona Clinic Liver Cancer, *HBV* Hepatitis B virus, *AFP* Alpha Fetoprotein

^a^Prior target therapy included sorafenib (*n* = 22), donafenib (*n* = 1) and apatinib (*n* = 1). Donafenib is a deuterated derivative of sorafenib. Apatinib is a multitarget tyrosine kinase inhibitor

Post-progression treatments were applied in 5 patients by PD-1 inhibitors and 10 by another targeted therapy in cohort 1. In Cohort 2, 4 patients received PD-1 inhibitors and 8 received other targeted agents.

### Primary and secondary endpoints

**Fig. 2:**
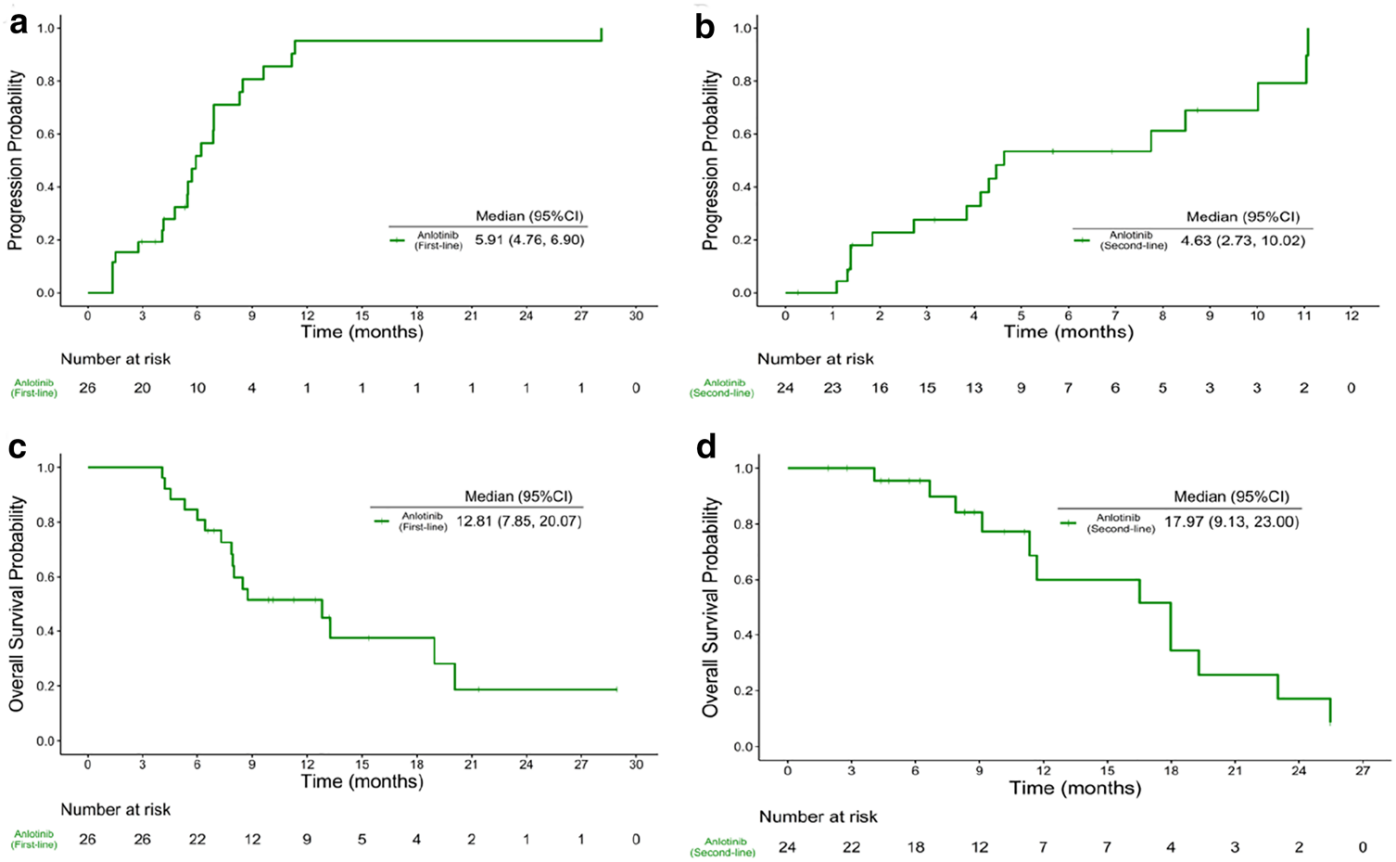
Kaplan–Meier time to progression curves of **a** Cohort 1 and **b** Cohort 2. Kaplan–Meier overall survival curves of **c** Cohort 1 and **d** Cohort 2

For patients in Cohort 1, the 12-week PFS rate was 80.8% (95% CI 59.8–91.5%), and 24-week PFS rate was 54.2% (95% CI 32.4–71.7%). The median TTP was 5.9 months (95% CI 4.8–6.9) and median OS was 12.8 months (95% CI 7.9–20.1) (Fig. 2a, c). One patient (3.9%) achieved a partial response (PR) and 21 patients (80.8%) had stable disease (SD) (Table 2).

**Table 2:** Primary and secondary endpoints

	Cohort 1 (*n* = 26)	Cohort 2 (*n* = 24)
12-week PFS rate (95% CI)	80.8% (59.8%, 91.5%)	72.5% (48.7%, 86.6%)
24-week PFS rate (95% CI)	54.2% (32.4%, 71.7%)	46.6% (24.4%, 66.2%)
TTP, month, [Median (95% CI)]	5.9 (4.8, 6.9)	4.6 (2.7, 10.0)
OS, month, [Median (95% CI)]	12.8 (7.9, 20.1)	18.0 (9.1, 23.0)
ORR (n)	3.9% (1)	8.3% (2)
Assessment of RECIST 1.1		
CR (%)	0	0
PR (%)	3.9% (1)	8.3% (2)
SD (%)	80.8% (21)	66.7% (16)
PD (%)	15.4% (4)	20.8% (5)
NE (%)	0	4.2% (1)

*PFS* progression-free survival, *TTP* time to progression, *OS* overall survival, *ORR* objective response rate, *RECIST* response evaluation criteria in solid tumors, *CR* complete response, *PR* partial response, *SD* stable disease, *PD* progressive disease, *NE* not be evaluated

With regards to the patients in Cohort 2, the 12-week PFS rate was 72.5% (95% CI 48.7–86.6%) and 24-week PFS rate was 46.6% (95% CI 24.4–66.2%). The median TTP and OS were 4.6 months (95% CI 2.7–10.0) and 18.0 months (95% CI 9.1–23.0), respectively (Fig. 2b, d). There were 2 patients (8.3%) who achieved a PR, and 16 (66.7%) achieved a SD (Table 2).

Further analysis of the OS showed, in Cohort 1, the median OS of the subgroups with and without subsequent targeted treatment were 20.1 months (95% CI 12.8–NE) and 7.9 months (95% CI 5.7–19.0) [*p* = 0.0160; hazard ratio (HR) = 0.26, 95% CI 0.08–0.84], respectively. However, the difference between subgroups of those with and without subsequent targeted treatment in Cohort 2 was not statistically significant [19.3 months (95% CI 18.0–23.0) vs. 11.7 months (95% CI 7.9–18.0), *p* = 0.3010]. With regard to the patients in Cohort 2, the median OS, redefined as from the initiation of the prior first-line targeted therapy to death of any cause, was 26.7 months (95% CI 20.6–36.9).

### Cytokine analysis

There were 18 baseline plasma samples available in Cohort 1 and 20 in Cohort 2. The number of matched plasma samples at baseline and at progression was both 15 in the two cohorts.

Comparing the concentrations at PD with those at baseline, several cytokines had increased [VEGF-A and C–C motif chemokine ligand (CCL) 11] or decreased [Programmed death ligand-2 (PD-L2) and CCL5] in both cohorts. The concentration of CXCL1 significantly increased by 2.8 ng/μl (*p* = 0.027) in Cohort 2. However, in Cohort 1, it increased by 3.5 ng/μl (*p* = 0.203). The change of FGF-2 concentration was not significant in either cohort. Neither did the baseline level of CXCL1 and FGF-2 between the two cohorts. (Table 3).

**Table 3:** Differences between baseline and disease progression cytokine concentration changes

	Cohort 1			Cohort 2		
Name of Cytokine	*N*^§^	Change (progression-baseline)	*p *value	*N*^§^	Change (progression-baseline)	*p *value
IL-2	8	− 16.6 (− 23.1, − 6.9)	0.0234	7	− 10.7 (− 34.5, − 1.0)	0.2969
IL-18	15	− 7.9 (− 19.5, 2.5)	0.0181	15	0.6 (− 16.0, 9.7)	0.8469
CCL11	15	9.8 (− 3.6, 17.8)	0.0353	15	7.5 (0.2, 16.4)	0.0413
CXCL1	9	3.5 (− 2.2, 6.8)	0.2031	9	2.8 (1.2, 20.6)	0.0273
CCL5	15	− 25.6 (− 34.0, − 15.0)	0.0001	15	− 12.6 (− 32.4, − 2.8)	0.0302
FGF-2	5	3.5 (− 3.5, 5.5)	1.0000	4	7.4 (2.4, 16.0)	0.1250
SCF	15	− 1.8 (− 4.2, 1.4)	0.0946	15	− 3.5 (− 7.0, − 1.4)	0.0026
VEGF-A	15	116.8 (70.9, 301.9)	0.0002	15	197.8 (8.1, 446.5)	0.0015
VEGF-D	15	− 1.0 (− 9.7, 3.4)	0.2958	15	0 (− 1.3, 9.9)	0.1882
PD1	15	− 7.5 (− 15.3, − 1.9)	0.0009	15	− 5.5 (− 12.0, 6.1)	0.4543
PD-L1	15	0.5(− 1.4, 2.3)	0.4459	15	0.9 (− 1.7, 1.8)	0.6100
PD-L2	15	− 160.0 (− 394.8, − 60.0)	0.0023	15	− 164.0 (− 289.0, − 105.0)	0.0067
CD137(4-1BB)	15	− 4.4 (− 15.6, 0)	0.0269	15	− 18.7 (− 25.6, 9.8)	0.1514

^§^The number of matched plasma samples, in which the concentration of cytokines could be detected efficiently

*IL-2* Interleukin-2, *IL-18* Interleukin-18, *CCL11* C–C motif chemokine ligand 11, *CXCL1* C-X-C motif chemokine ligand 1, *CCL5* C–C motif chemokine ligand 5, *FGF-2* Fibroblast growth factor, *SCF* Stem cell factor, *VEGF-A* Vascular Endothelial Growth Factor-A, *VEGF-D* Vascular Endothelial Growth Factor-D, *PD-1* Programmed Death-1, *PD-L1* Programmed Death Ligand-1, *PD-L2* Programmed death ligand-2

CXCL1 was markedly correlated with TTP in both cohorts at the cutoff value of 7.6 ng/μl, the median concentration in Cohort 2. The median TTP of Cohort 1 patients in whom the level of CXCL1 was more or less than 7.6 ng/μl were 5.9 months (95% CI 1.4–6.9) and 9.1 months (95% CI 6.9–11.2), respectively, *p* = 0.0071, while that in Cohort 2 was 2.8 months (95% CI 1.1–7.8) and 10.0 months (95% CI 2.7–11.7), respectively, *p* = 0.0029. (Supplementary Fig. 1) There was no significant correlation between the plasma CXCL1 level and efficacy at the cutoff value selected by ROC analysis. Neither was the relationship between the concentration of other cytokines and TTP or OS.

### Adverse events

In general, adverse events of anlotinib were tolerable. The most common AEs were hypertension and hypothyroidism (both 62%, 31/50), followed by fatigue (56%), hand-foot syndrome (54%), elevated bilirubin (52%) and diarrhea (52%). AEs at grade 3 or higher were mainly hypertension, diarrhea and hand-foot syndrome (8%, 8% and 6%, respectively) (Table 4). In this study, 2 of 5 serious AEs were probably anlotinib related, which were both grade 3 gastrointestinal bleeding. After symptomatic treatment, one recovered and the other died.

**Table 4:** Most common adverse events (incidence > 5%) and the adverse events ≥ Grade 3

	AE at any level, *n* (%)			AE ≥ level 3, *n* (%)		
	total	Cohort 1	Cohort 2	total	Cohort 1	Cohort 2
Hypertension	31 (62.0%)	19 (73.1%)	12 (50.0%)	4 (8.0%)	3 (11.5%)	1 (4.2%)
Hand-foot syndrome	27 (54.0%)	14 (53.8%)	13 (54.2%)	3 (6.0%)	2 (7.7%)	1 (4.2%)
Elevated bilirubin	26 (52.0%)	15 (57.7%)	11 (45.8%)	2 (4.0%)	1 (3.8%)	1 (4.2%)
Fatigue	28 (56.0%)	16 (61.5%)	12 (50.0%)	–	–	–
Proteinuria	25 (50.0%)	13 (50.0%)	12 (50.0%)	1 (2.0%)	–	1 (4.2%)
Diarrhea	26 (52.0%)	16 (61.5%)	10 (41.7%)	4 (8.0%)	4 (15.4%)	–
Elevated Alanine Aminotransferase	9 (18.0%)	4 (15.4%)	5 (20.8%)	1 (2.0%)	1 (3.8%)	–
Hoarseness	11 (22.0%)	6 (23.1%)	5 (20.8%)	–	–	
Hypertriglyceridemia	13 (26.0%)	7 (26.9%)	6 (25.0%)	–	–	–
Hypercholesterolemia	10 (20.0%)	5 (19.2%)	5 (20.8%)	–	–	–
Elevated aspartate aminotransferase	14 (28.0%)	9 (34.6%)	5 (20.8%)	1 (2.0%)	1 (3.8%)	–
Anorexia	18 (36.0%)	10 (38.5%)	8 (33.3%)	–	–	–
Bleeding	13 (26.0%)	10 (38.5%)	3 (12.5%)	2 (4.0%)	2 (7.7%)	–
Leukopenia	17 (34.0%)	8 (30.0%)	9 (37.5%)	–	–	–
Thrombocytopenia	18 (36.0%)	8 (30.0%)	9 (37.5%)	–	–	–
Abdominal pain	20 (40.0%)	10 (38.5%)	10 (41.7%)	2 (4.0%)	1 (3.8%)	1 (4.2%)
Hypothyroidism	31 (62.0%)	16 (61.5%)	15 (62.5%)	–	–	–
Prolonged QT interval	18 (36.0%)	10 (38.5%)	8 (33.3%)	1 (2.0%)	1 (3.8%)	–
Elevated low-density lipoprotein	16 (32.0%)	9 (34.6%)	7 (29.2%)	–	–	–
Nausea	4 (8.0%)	3 (11.5%)	1 (4.2%)	–	–	–
Fever	3 (6.0%)	1 (3.8%)	2 (8.3%)	–	–	–
Hepatalgia	2(4.0%)	2 (7.7%)	–	–	–	–
Cough	9 (18.0%)	6 (23.1%)	3 (12.5%)	–	–	–
Vomit	6 (12.0%)	3 (11.5%)	3 (12.5%)	–	–	–
Anemia	3 (6.0%)	1 (3.8%)	2 (8.3%)	1 (2.0%)	–	1 (4.2%)
Weight loss	6 (12.0%)	5 (19.2%)	1 (4.2%)	–		–
Headache	6 (12.0%)	5 (19.2%)	1 (4.2%)	–	–	–
Chest tightness	4 (8.0%)	2 (7.7%)	2 (8.3%)	–	–	–
Elevated lipase in blood	6 (12.0%)	6 (23.1%)	–	1 (2.0%)	1 (3.8%)	–
Toothache	4 (8.0%)	4 (15.4%)	–	–	–	–
Pharyngalgia	23 (46.0%)	11 (42.3%)	12 (50.0%)	–	–	–
Low back pain	6 (12.0%)	4 (15.4%)	2 (8.3%)	1 (2.0%)	–	1 (4.2%)
Musculoskeletal pain	7 (14.0%)	4 (15.4%)	3 (12.5%)	–	–	–
Arthralgia	6 (12.0%)	3 (11.5%)	3 (12.5%)	–	–	–

## Discussion

In this study, anlotinib showed promising antitumor properties against HCC as a first-line treatment. In Cohort 1, anlotinib achieved a median TTP of 5.9 months and a 12-week PFS rate of 80.8%. These results seemed numerically better than sorafenib in Chinese patients, that the median TTP was 2.8 and 3.7 months in the Oriental and REFLECT studies, respectively [3, 16]. So far, lenvatinib has shown the longest median TTP of 7.4 months in first-line TKI treatment [16]. In IMbrave 150 trial, the ORR of atezolizumab combined with bevacizumab was 30%, the median PFS and OS were 6.9 months and 19.2 months, respectively. In Chinese cohort, the updated median OS of combination therapy achieved 24 months [9]. Whether there was a numerical difference between monotherapy, such as anlotinib or lenvatinib, and combination therapy, the difference should be validated in the future.

As expected, anlotinib appeared to have similar activity in HCC refractory to anti-angiogenic TKIs, with a median TTP of 4.6 months and a PFS rate at 12 weeks of 72.5%. In previous studies, anti-angiogenic-based second-line treatments showed a median PFS of 2.8, 3.1 and 5.2 months, a median OS of 8.5, 10.6 and 10.2 months with ramucirumab, regorafenib and cabozantinib, respectively. ICIs in the second-line setting showed a PFS of 2.3 to 4.9 months. The median OS of 18.0 months in Cohort 2 was even more encouraging. The broad inhibition of FGFR1-4 might be one of the underlying mechanisms of being active in HCC refractory to anti-angiogenic TKIs [17]. The difference between with or without subsequent TKI treatment was enormous, which further indicated that continuous anti-angiogenic therapies could bring more survival benefit for patients with advanced HCC. Based on the rich blood perfusion and continuous inhibition of angiogenesis strategy of treatment in HCC, the anti-angiogenic tyrosine kinase inhibitors (TKIs) would be chosen as second-line regimens, even under the circumstance of first-line anti-angiogenic agents combined with ICIs therapy.

We found patients with lower baseline plasma CXCL1 levels achieved significantly longer TTP. Furthermore, the level of CXCL1 increased significantly as the disease progressed in Cohort 2. CXCL1 is involved in the induction of neutrophils migration, angiogenesis, arteriogenesis, inflammation, wound healing and tumorigenesis. It was demonstrated that CXCL1, secreted from tumor cells, induced migration of vascular endothelial cells and angiogenesis in vitro*. *In vivo studies indicated that the tumor angiogenesis could be reduced by CXCL1 pathway blocking [18]. CXCL1 pathway might be involved in sorafenib resistance. In sorafenib-treated osteosarcoma and ovarian cancer, the expression of CXCL1 increased significantly [19, 20]. Blockade of CXCR2, the receptor of CXCL1, elevated the efficacy of sorafenib and delayed resistance [20]. The microRNA 30A and microRNA 200A, could regulate the expression of CXCL1, was observed to be related to the efficacy of regorafenib. [21–23]. Overall, we postulated that CXCL1 might be a critical angiogenic promotor and play an important role in both primary and secondary resistance to VEGF/VEGFR inhibitors in HCC. Those microRNAs related to CXCL1 expression might also be predictors for anlotinib, which could be verified in future studies.

Anti-angiogenesis of anlotinib could contribute to the regulation of tumor microenvironment and inflammation in tumors [24, 25]. In our study, the concentration of plasma VEGFA and CCL11 were significantly increased at PD, and level of PD-L2 and CCL5 were significantly decreased. The impact of these changes needs to be further illustrated.

Anlotinib has been investigated in a variety of malignant tumors [26–29]. In this study, there were no unexpected AEs. The most common grade 3 or 4 AEs of anlotinib included hypertension (8%), diarrhea (8%) and hand-foot syndrome (6%). In the REFLECT study, the most common grade 3 or 4 AEs of lenvatinib were hypertension (23%), weight loss (8%), proteinuria (6%), thrombocytopenia (5%), and liver dysfunction (5%) [16]. In the RESORCE study of regorafenib, the most common grade 3 or 4 AEs were hypertension (15%), hand-foot syndrome (13%), elevated AST (10%), elevated bilirubin (10%), and fatigue (9%) [4]. Thus, the incidence of AEs in anlotinib seemed lower than other standard TKIs in hepatocellular carcinoma. All grade 3 or 4 AEs occurred in less than 10% of patients. The incidence of grade 3 or 4 hypertension in anlotinib seemed lower than that in lenvatinib and regorafenib. Besides, the safety profile was similar between anlotinib and other TKIs. In a randomized phase II trial of anlotinib in comparison with sunitinib as first-line treatment in renal cell carcinoma, grade 3 or 4 AEs in anlotinib were significantly less frequent than that in sunitinib (28.9% versus 55.8%, *p* < 0.01), especially in terms of thrombocytopenia and neutropenia [29].

Taken the safety profile together, anlotinib could be one of the potential partners for combination therapy with ICIs as first-line treatment in hepatocellular carcinoma. Exploratory trials of anlotinib with ICIs have been conducted already. A phase II trial (NCT04172571) aimed to evaluate the safety and efficacy of first-line anlotinib plus penpulimab, an IgG1 anti-PD-1 antibody, in which the preliminary ORR was 31% with acceptable toxicity [30]. We had begun another phase II trial (NCT03825705) to assess the safety and efficacy of anlotinib combined with TQB2450, an IgG1 anti-PD-L1 antibody, and had observed preliminary efficacy (unpublished).

This was an open-label phase II clinical study. The sample size of the two cohorts was limited. Only the preliminary efficacy and safety data of HCC treatment could be provided, which still needed to be further verified by expanded clinical research. More clinical data were needed to verify the predictive role of CXCL1. Some patients in both the cohorts received ICIs, which interfered with the interpretation of OS results.

## Conclusion

Anlotinib showed promising and similar activities both in the first- and second-line treatment of advanced HCC. Anlotinib was well tolerated and is expected to be a favorable partner of ICIs. The role of anlotinib in advanced HCC warrants further validation in future clinical trials.

## Supplementary Information

Below is the link to the electronic supplementary material.

Supplementary file1 (DOCX 31 kb)

## Declarations

### Availability of data and material

The data that support the findings of this study are available from the corresponding author upon reasonable request.
