# Supplementary material for: Anlotinib in the treatment of advanced hepatocellular carcinoma: an open-label phase II study (ALTER-0802 study)
Source: Hepatol Int. 2021 Apr 7;15(3):621–9. doi: 10.1007/s12072-021-10171-0 (PMC8286948; doi:10.1007/s12072-021-10171-0)

**Supplementary materials**

Supplementary Table 1

| IFN-gamma | IL-17A | TNF-beta | EGF | IDO |
| --- | --- | --- | --- | --- |
| IL-12p70 | IL-21 | CCL11 | FGF-2 | LAG-3 |
| IL-13 | IL-22 | CXCL1 | HGF | PD1 |
| IL-1beta | IL-23 | IL-8 | LIF | PD-L1 |
| IL-2 | IL-27 | IP-10 | PDGF-BB | PD-L2 |
| IL-4 | IL-9 | MCP-1 | PlGF-1 | TIM-3 |
| IL-5 | IFN-alpha | MIP-1alpha | SCF | CD28 |
| IL-6 | IL-31 | MIP-1beta | VEGF-A | CD80 |
| TNF-alpha | IL-15 | SDF-1alpha | VEGF-D | CD137(4-1BB) |
| GM-CSF | IL-1alpha | CCL5 | BTLA | CD27 |
| IL-18 | IL-1RA | NGF-beta | GITR | CTLA-4 |
| IL-10 | IL-7 | BDNF | HVEM |  |

## IFN, Interferon; IL, Interleukin; TNF, Transforming Growth Factor; GM-CSF, Granulocyte-macrophage Colony Stimulating Factor; CCL, C-C motif chemokine ligand; CXCL, C-X-C motif chemokine ligand; MCP, [Monocyte chemotactic protein](http://www.dictall.com/indu/348/34724923024.htm); MIP, Macrophage inflammatory protein; SDF, Stromal cell derived factor; FGF, Fibroblast Growth Factor; NGF, Nerve growth factor; BDNF, Brain-derived neurotrophic factor; EGF, Epidermal growth factor; FGF, Fibroblast Growth Factor; HGF, Hepatocyte Growth Factor; LIF, Leukemia inhibitory factor; PDGF, Platelet-derived Growth Factor; SCF, Stem cell factor; VEGF, Vascular Endothelial Growth Factor-A; BTLA, B and T lymphocyte attenuator; GITR, glucocorticoid-induced TNF receptor; HVEM, herpesvirus entry mediator; IDO, indoleamine-2,3-dioxygenase; LAG-3, lymphocyte activation gene 3; PD-1, Programmed Death-1; PD-L1, Programmed Death Ligand-1; PD-L2, Programmed Death Ligand-1; TIM-3, T cell immunoglobulin and mucin domain 3; CTLA-4, Cytotoxic T lymphocyte-associated Antigen-4.

Supplementary Figure 1 The Kaplan-Meier overall survival curve of according to CXCL level in (A) cohort 1 and (B) cohort 2.
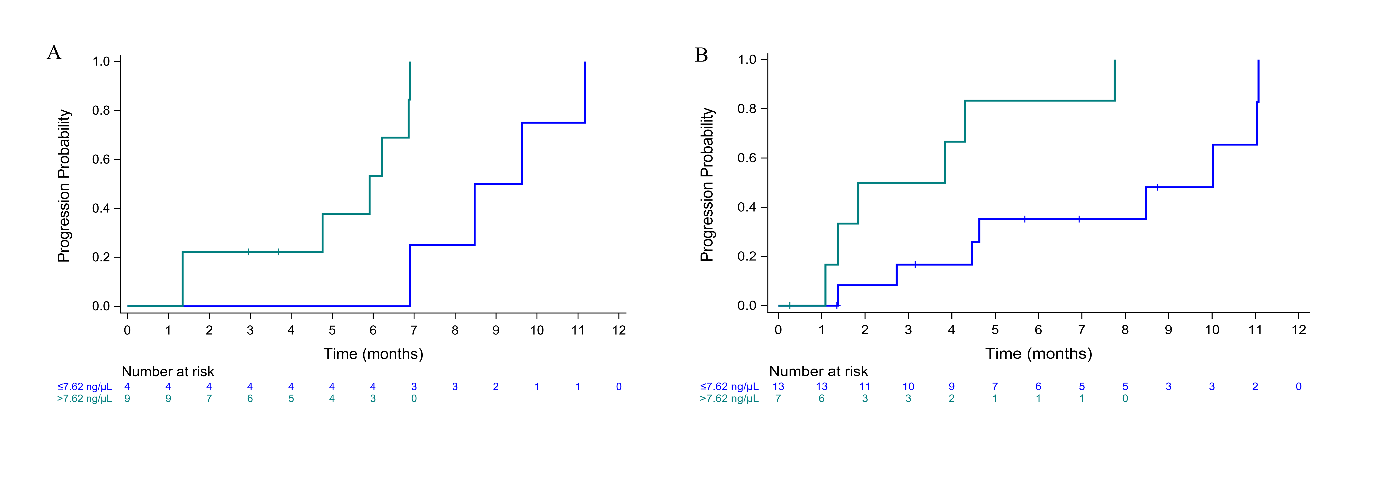

Supplement: Supplementary file 1 — Supplementary file1 (DOCX 31 kb) [file 12072_2021_10171_MOESM1_ESM.docx]
